# Endometrial Stromal Cells Circulate in the Bloodstream of Women with Endometriosis: A Pilot Study

**DOI:** 10.3390/ijms20153740

**Published:** 2019-07-31

**Authors:** Júlia Vallvé-Juanico, Carlos López-Gil, Agustín Ballesteros, Xavier Santamaria

**Affiliations:** 1 Department of Reproductive Medicine, IVIRMA-Barcelona S.L., 08017 Barcelona, Spain; 2 Group of Biomedical Research in Gynecology, Vall Hebron Research Institute (VHIR) and University Hospital, 08035 Barcelona, Spain; 3 Center for Reproductive Sciences, Department of Obstetrics, Gynecology and Reproductive Sciences, University of California, San Francisco, CA 94143, USA; 4 Igenomix Foundation, Paterna, 46980 Valencia, Spain

**Keywords:** endometriosis, circulating endometrial cells, CD10, stromal cells, diagnostics, liquid biopsy

## Abstract

Endometriosis is characterized by the presence of endometrial tissue outside the uterus. While endometriotic tissue is commonly localized in the pelvic cavity, it can also be found in distant sites, including the brain. The origin and pathophysiology of tissue migration is poorly understood; retrograde menstruation is thought to be the cause, although the presence of endometrium at distant sites is not explained by this hypothesis. To determine whether dissemination occurs via the bloodstream in women with endometriosis, we analyzed circulating blood for the presence of endometrial cells. Circulating endometrial stromal cells were identified only in women with endometriosis but not in controls, while endometrial epithelial cells were not identified in the circulation of either group. Our results support the hypothesis that endometrial stromal cells may migrate through circulation and promote the pathophysiology of endometriosis. The detection of these cells in circulation creates avenues for the development of less invasive diagnostic tools for the disease, and opens possibilities for further study of the origin of endometriosis.

## 1. Introduction

Endometriosis is a benign disease characterized by the growth of endometrial tissue outside of the uterine cavity which affects approximately 15% of pre-menopausal women [1,2]. The main symptoms of the disease are chronic pelvic pain, dysmenorrhea, dyspareunia and infertility/subfertility. All these symptoms might affect patients psychologically, which often has repercussions on their social lives [3]. 

Endometriotic lesions are typically located in the pelvic region; however, distant lesions have been reported in lung, liver, and even in the eyes [4]. Several explanations for the origin of endometriosis have been proposed, but no clear consensus has been reached. The most accepted explanation is Sampson’s hypothesis, which describes retrograde menstruation [5,6]. However, this theory has some limitations; for example, the 95% of women have retrograde menstruation, while only the 15% manifest the disease [7]. Therefore, other explanations, such as coelomic metaplasia or embryonic rests, have also been proposed [5,7]. Nonetheless, none of these hypotheses account for cases of distant lesions identified outside the pelvis. Alternatively, bone marrow or endometrial stem cells could play a role in the origin of endometriosis, as shown in some studies where bone marrow stem cells engrafted endometriotic lesions [8,9,10,11]. Moreover, rodent models of endometriosis demonstrated that endometriotic cells migrated from lesions to eutopic endometrium [12], suggesting that they migrated through extravasation. Interestingly, this process seems to be regulated by an epithelial-mesenchymal transition (EMT), since the migrating cells express aberrant epithelial markers in the stroma [12]. EMT is an important process in metastasis, as cells are able to change their phenotype from epithelial to a mesenchymal type, which allows the extravasation of the cells, enabling them to migrate to other tissues. Additionally, hematogenous dissemination of mesenchymal stem cells occurs in rodent models of endometriosis, suggesting the presence of circulating endometriotic cells [13]. The aberrant expression of epithelial markers in the stromal compartment of the eutopic endometrium has also been demonstrated in humans [14] by our group. Epithelial cells, expressing cytokertin (CK) and E-Cadherin, were found in the stromal layer of eutopic endometrium co-expressing the putative stem cell marker LGR5 [14,15]. In our study, EMT could play a role in the migration of endometrial LGR5^+^CK^+^ cells, since they also overexpressed MMP12, a matrix metalloproteinase involved in the degradation of the extracellular matrix and involved in the EMT process [16]. Taken together, these findings suggest that endometrial cells might be able to migrate from eutopic endometrium to ectopic sites through circulation thanks to an EMT process. 

Endometriosis is generally diagnosed by laparoscopy, an invasive procedure, with subsequent histopathologic assessment of tissue samples. Therefore, new and less invasive approaches would aid the diagnostic process. Although attempts have been made to identify biomarkers in the eutopic endometrium, in serum or plasma, in peritoneal fluid, and in urine [17], no biomarkers to date demonstrate adequate specificity or sensitivity. 

Liquid biopsy is a novel technique that consists of detecting and isolating circulating tumor cells as source for genomic information for cancer patients enabling a potential non-invasive diagnosis. 

Indeed, the identification of circulating cells in blood has been used for the diagnosis of cancers such as colon, prostate, renal, pancreatic, and lung cancer [18,19,20,21,22]. Circulating tumor cells (CTCs) are shed into the bloodstream from primary or metastatic tumors, and have the potential to initiate metastasis in distant tissues or organs [23,24]. Cancer cells are shed from the primary tumor into the circulation prior to the presentation of clinical symptoms and may metastasize at distant sites [25], sparking interest in the development of new tools using liquid biopsy for CTCs isolation. Numerous methods have been developed to detect CTCs, primarily based on the detection of epithelial cell adhesion molecule (EpCAM). The Food and Drug Administration (FDA) has approved the CellSearch^®^ platform for the detection of CTCs, and it is being used successfully to detect them in breast cancer [26,27,28,29]. However, not all cancers or diseases shed circulating cells with epithelial origin into the bloodstream. Moreover, EMT seems to be common among circulating cells, and they may lose their epithelial markers, making their detection in blood much more difficult [30,31]. Therefore, alternative methods to identify not only epithelial, but also stromal cells, need to be assessed. 

The aim of our study was to evaluate the presence and nature of circulating cells in women with endometriosis through liquid biopsy using a user friendly circulating rare cells (CRCs) isolation device based on size exclusion to capture not only epithelial, but also stromal circulating cells in blood. For this reason, in order to evaluate the phenotype of circulating cells, we labeled the obtained cells with the epithelial and stromal markers cytokeratin (CK) and CD10, respectively.

## 2. Results

### Isolation and Detection of Circulating Endometrial Cells (CECs): Immunofluorescence

We found a mean of 14.75 circulating endometrial cells (CECs; range 0–57) in the group with endometriosis (*n* = 8) and no CECs in the four healthy donors. The counts of total cells present in the total area of the filter are listed in Table 1. 

**Figure 1:**
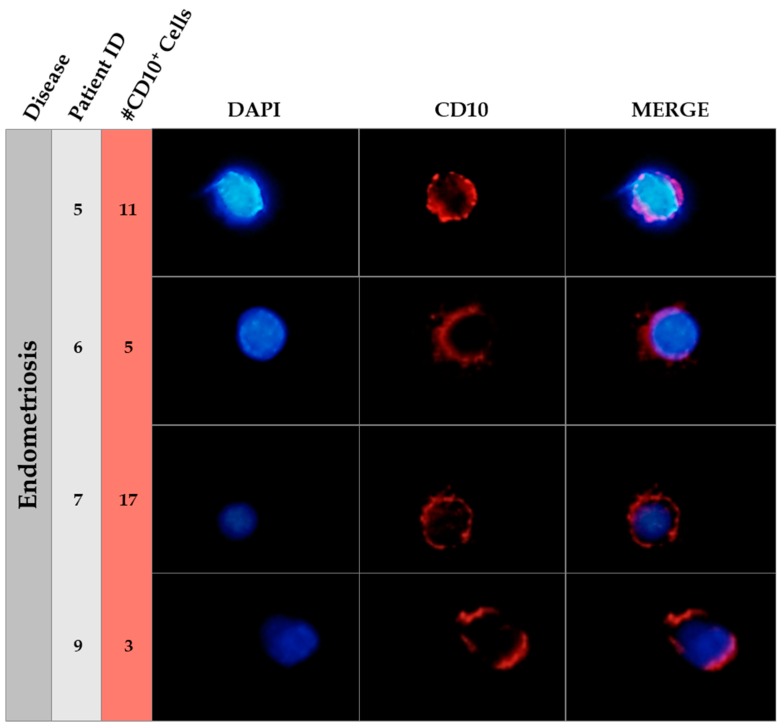
Immunofluorescence of CD10 in endometriosis patients. This figure shows an example of the CD10 positive cells found in circulating blood of endometriosis patients. The patient ID is showed in the second column and the number of CD10 positive cells in the third column. Nuclear staining (DAPI, blue), CD10 positive cells (Alexa555, Red) and merge pictures are shown for each patient. The field was taken at ×40 magnification. A manual amplification of a CD10 positive cell in each case is shown in the figure.

Surprisingly, we observed CECs expressing the endometrial stromal marker CD10, also called CALLA (common acute lymphoblastic leukemia antigen), but did not find any cytokeratin (CK) positive cells in the endometriosis group nor in the control group. In addition, no cells expressing CD10 displayed co-localization with the CK marker. An example of the presence of CD10^+^ cells in circulation in each of the four patients with endometriosis where the marker was found is shown in Figure 1.

Interestingly, the only instance where no CEC were found (patient 8) was a patient with deep infiltrating endometriosis (DIE) who had been under GnRH agonist suppression treatment for two months before sample collection. Moreover, another patient who had DIE (patient 9) underwent surgery four months before the sample collection, and only three CECs expressing CD10 were found in circulation when she was under hormone replacement therapy (HRT) with GnRH downregulation. Blood from patient 9 was taken at three additional time points (during the proliferative, secretory, and menstrual phases); surprisingly, CD10^+^ cells were found in all cases. However, when she was under GnRH treatment, only three CD10^+^ cells were found in circulation, whereas when the agonist was removed, 57 CD10^+^ cells were found during the proliferative phase of the menstrual cycle and 22 CD10^+^ cells during the secretory phase.

No differences in the number of CECs across the phases of the menstrual cycle were observed, nor were there any differences among different ages of women. Indeed, in patient 9, no differences between cycle phases were observed. 

## 3. Discussion 

Here, we demonstrate for the first time that the ScreenCell^®^ device may be used to isolate CECs from peripheral blood in women with endometriosis, even with the very small amount of CECs that are normally present in a liquid biopsy. CD10^+^ cells (stromal cells) were present in the peripheral blood of women with endometriosis, but not epithelial cells. ScreenCell^®^ Cyto Technology isolates circulating cells based on size exclusion. An advantage of this device is its rapid processing time; the assay can be completed in about 15 min. A series of 23 patients with cutaneous melanoma, a cell type that does not express EpCAM, revealed the utility of the ScreenCell^®^ Cyto device for enumeration of CTCs, cytological analysis, and analysis of genetic mutations [32].

We used CD10 to identify stromal endometrial circulating cells, since it is a marker expressed by endometrial stromal cells in normal endometrium as well as in the stromal compartment of endometrial neoplasms [33], and in ectopic endometriotic lesions [34]. The presence of CD10 has also been observed in some B and T lymphocytes in patients with leukemia [35,36]. However, none of the tested patients had leukemia. In addition, CD10^+^ endometrial cells are usually larger than hematopoietic cells and most of the cells pass through the filter while circulating CECs are retained. Indeed, the filter pores measure 7.5–8 µm in diameter and are able to retain 85–100% of “tumor” or endometrial cells and only 0.1% of common blood cells [32]. 

We found stromal cells (CD10^+^ cells) but no epithelial cells in the circulating blood of endometriosis patients. One explanation for the absence of epithelial circulating cells may be that cells smaller than 8 µm could be missed using this filtration technique. Alternatively, epithelial cells undergo EMT when they spread to the blood circulation and do not express CK. Invasive cancer cells migrate through the stroma and vessels as single cells or in clusters and, while single cells usually undergo EMT, clusters harbor mesenchymal cells [37], suggesting that EMT plays an important role in the initiation of metastasis [36]. Even though endometriosis is a benign disease, it is believed that mesenchymal stem cells could be responsible for the endometriotic lesions observed in organs far from the endometrial cavity [13]. Our findings support this hypothesis, as we found mesenchymal circulating cells only in patients with endometriosis but not in healthy patients. Since it was not possible to determine the origin of the stromal cells found in blood circulation, it is not clear if these stromal cells migrate through extravasation from the eutopic endometrium or, on the other hand, from the endometriotic lesion, enhancing the spread of the disease to distant sites. Alternatively, it is known that tissues derived from coelomatic epithelial and mesenchymal cells can differentiate into epithelial and stromal cells [7] and the environment in peritoneal cavity of women with endometriosis is altered as well [38]. Therefore, an unproven but plausible explanation could be that immune cells, pro-inflammatory cytokines/chemokines, adhesion molecules, among other factors, could activate the differentiation of quiescent endometrial progenitor stem cells into endometrial cells and lead to the development of endometriotic lesions during pubertal development [7]. 

Several efforts have been made for the detection of CTCs in gynecological cancers by using different approaches. However, to our knowledge, only two groups have investigated circulating cells in peripheral blood in women with endometriosis [39,40]. A summary of the approaches performed in different gynecological diseases is shown in Table 2.

In the case of endometriosis, one group used MetaCell^®^ Technology, in which cells are enriched by filtration. CECs were cultured and underwent immunohistochemistry (IHC) with CK, vimentin, and CD10 markers to confirm endometrial origin [39]. CECs were identified in 4/17 cases of endometriosis. In this study, we used ScreenCell^®^ device, which is also based in size exclusion method, therefore, the loosing or lack of epithelial markers on the cell surface is solved and all types of circulating cells in blood can be captured. One advantage of the ScreenCell^®^ device when compared to MetaCell^®^ is that it is able to fix and label the cells directly into the filter without the need to culture them, ensuring that the surface markers do not change during the processing of the sample. The second group used microfluidic chips and found that 89.5% of endometriotic patients had circulating CECs, while only 15% of the healthy controls had circulating CECs [40]. They identified CECs by positive expression of the vimentin/CK and estrogen/progesterone receptor. We obtained CECs in all endometriotic patients except one (4 out of 5 samples). Interestingly, the only patient in which CECs were not detected had been under agonist (GnRH) suppression for two months after laparoscopic intervention. The first time that blood samples were obtained from this patient was during the proliferative phase underHRT treatment after GnRH downregulation. At this time, only three CD10^+^ cells were found in circulation. The second collection time was during the menstrual phase, right before starting an HRT cycle and without agonist treatment. After that, two more samples were taken at the proliferative and secretory phase during HRT treatment. Surprisingly, in the last cycle without GnRH downregulation, the number of positive stromal cells in circulation increased considerably; 57 and 22 CD10^+^ cells were found, respectively. These findings suggest that CECs might be an indicator of disease activity, since some studies have demonstrated that CTCs are correlated with disease progression [54]. We included patients with controlled ovarian stimulation (COS) and HRT and, according to our data, the type of endometrial preparation does not seem to affect to the number of CECs found. 

A strength of this study is that we analyzed fixed cells directly on the membrane of the filter, indicating that the cells did not have a chance to change their morphology or their gene expression. In addition, another advantage when using ScreenCell^®^ device is that it is a very friendly user device, which is easy to manipulate and the whole filtration process is very fast, obtaining the CECs in less than 15 min. On the other hand, this study presents several limitations. First, the sample size is small, and samples were obtained in different phases of the menstrual cycle. However, Chen et al. did not find any significant difference among menstrual phases when they tested 57 patients [40], also suggesting that phases of the cycle do not affect to the number of CECs. Another limitation is that none of the control patients underwent laparoscopy. Instead, we included proven fertile 18–35 year-old donors with no signs or symptoms of endometriosis.

In conclusion, we have identified stromal endometrial cells in circulating blood from women with endometriosis, providing evidence to assist the development of new and less invasive tool for the early diagnosis of endometriosis. In addition, our results suggest that stromal cells could be involved in the pathophysiology of endometriosis by migrating through circulation to distant sites. However, further studies are required to assess the feasibility of utilizing CECs in the diagnosis of endometriosis and to investigate the nature of endometrial cell migration.

## 4. Material and Methods

### 4.1. Subjects

**Figure 2:**
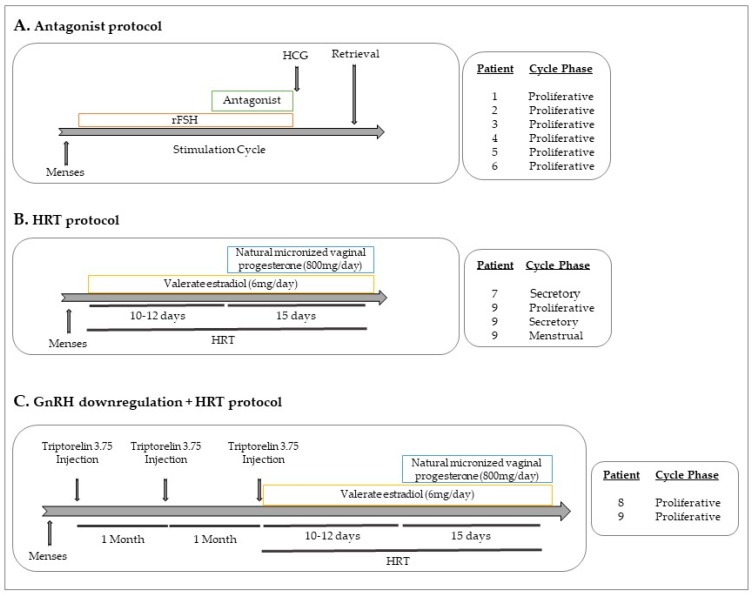
Hormonal treatments. (**A**) Antagonist protocol: After menses, rFSH is administrated for around 10–12 days. When the follicles reach around 12mm, the antagonist (GnRHAn) is administrated. At the end of the stimulation cycle, hCG is injected before the oocyte retrieval. (**B**) HRT protocol: After menses, valerate estradiol at oral dosages (6–8 mg/day) for 10–12 days is administrated. Then, 800 mg/day of natural micronized progesterone vaginally are added to the estradiol treatment for 15 more days. (**C**) GnRH downregulation protocol + HRT: in luteal phase, one injection of triptorelin 3.75 (GnRHa) is administrated and subsequently for two more months. rFSH: recombinant follicle stimulating hormone; GnRHa: gonadotropin releasing hormone agonist; GnRHAn: gonadotropin releasing hormone antagonist; HRT: hormonal replacement treatment; hCG: human chorionic gonadotropin.

Samples were collected between June 2017 and January 2018 at IVI Barcelona S.L. and Hospital Vall Hebron (Barcelona, Spain) and all participants signed an informed consent. Nine participants were included in the study: four healthy women who were egg donors and five patients with endometriosis diagnosed by laparoscopy. None of the participants had previous records of oncological pathologies. From the five endometriosis patients, two were stimulated with recombinant follicular stimulating hormone (rFSH), in an antagonist cycle (patients 5 and 6). Three were under HRT cycle (patients 7, 8 and 9) and two of them with previous GnRH downregulation (patients 8 and 9). Briefly, HRT consisted of estradiol at oral dosages of 6–8 mg/day for 10–12 days and then adding 800 mg/day of natural micronized progesterone vaginally. Patient 9 donated blood at four different time points after surgery: while she was under HRT treatment (at the proliferative phase), while under GnRH downregulation, and only with HRT treatment (during the proliferative, secretory and menstrual phases of the cycle). Figure 2 shows the protocols of the different treatments in patients with endometriosis. 

All participants were pre-menopausal and aged between 18 and 44. The mean age of study participants was 30.77 years (range 21–42). The mean age in the control group was 25 years (range 21–31) and 35.4 years (range 30–42 years old) in the endometriosis group. Patient characteristics are listed in Table 1. 

Among the five endometriotic patients, three were diagnosed with DIE (patients 5, 8, and 9) and the other two presented ovarian endometriomas (patients 6 and 7). Patient 6 manifested a 3 cm endometrioma in the right ovary and a 2 cm endometrioma in the left ovary, whereas patient 7 had a 2 cm endometrioma in the right ovary. Among the patients with DIE, patient 5 presented bowel and vesical lesions and a 2 cm adenomyoma as well. Patient 8 showed pelvic, utero-sacral and bowel lesions and multiple infracentimetric endometriotic lesions in both ovaries (stage IV), whereas patient 9 presented multiple bowel and pelvic adhesions together with a 2 cm endometrioma in the right ovary (stage IV). Regarding reproductive outcomes, two patients had previously conceived healthy children (patients 6 and 8) after assisted reproductive technology (ART) treatments, while the other three had never been pregnant.

Two samples from two different patients were obtained during the mid-late follicular phase of COS with antagonist (patients 5 and 6) and the rest of the samples from the endometriotic group were obtained in women undergoing HRT for a frozen embryo transfer. Three of the samples from two different patients were obtained during the proliferative phase. Two samples from two different patients (patients 7 and 9) were obtained in the secretory phase and one in the menstrual phase of the cycle (patient 9). Among the patients undergoing HRT, two of the samples (from patients 8 and 9) were obtained after GnRH agonist down-regulation (one after three months of GnRH down regulation and the other after only one month of GnRH downregulation). 

### 4.2. Sample Collection

Twelve blood samples of 10 mL were obtained in K2-EDTA tubes (Becton Dickinson, Franklin Lakes, NJ, USA). Proven healthy donor samples were collected during COS. To avoid possible cutaneous contamination from epithelial cells taken by the needle during sampling, the first milliliter of blood obtained was always discarded. After blood collection, tubes were immediately inverted ten times and kept at 4 °C until they were processed. The ethics committee of IVI Barcelona S.L. (1611-BCN-080-XS; 6 June 2016) and Vall Hebron (PR(AMI)410/2016; 7 July 2017) approved the use of the blood samples. 

### 4.3. Sample Processing

**Figure 3:**
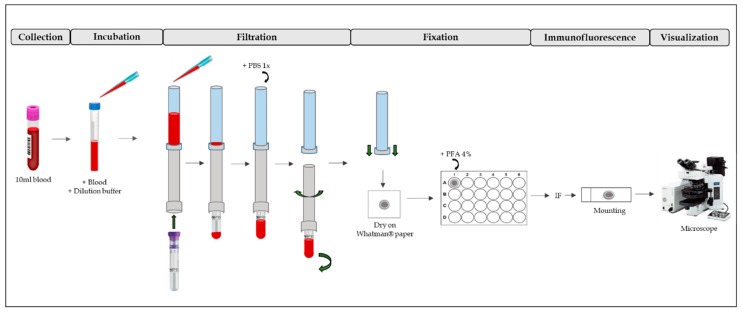
ScreenCell^®^Cyto Technology process. First, blood is collected and mixed with dilution buffer (Incubation). After, there is a filtration process, washing and fixation with PFA 4%. Then, IF can be performed on the filter. After mounting, the IF is visualized by a fluorescence microscope. PBS: phosphate buffered saline; PFA: paraformaldehyde; IF: immunofluorescence.

To obtain circulating endometrial cells (CECs), we utilized ScreenCell^®^ Cyto Technology (ScreenCell^®^Cyto CY 4FC; ScreenCell SA, Paris, France). We followed the manufacturer’s instructions, briefly outlined below and in Figure 3. 

First, the ScreenCell^®^ FC dilution buffer was prepared. After that, 3 mL of blood were transferred into one 15 mL sterile conical tube. Then, 4 mL of dilution buffer was added and inverted five times and incubated for 8 min at room temperature (RT). During this process, red blood cells became lysate. After incubation, the blood was filtered. The filter contains pores of different sizes (6.5 ± 0.33 µm) that are randomly distributed around the entire filter surface. Then, 1.6 mL of 1× phosphate buffered saline (PBS) was added to wash the filter of debris. The filter was then placed on a piece of Whatman^®^ paper to be dried. After, it was placed it in a p24 well and fixed with 4% paraformaldehyde for 10 min. It was then washed three times for 5 min with 1× PBS. Finally, immunofluorescence was performed. 

### 4.4. Detection of CECs: Immunofluorescence

To determine whether CECs were of epithelial or stromal origin, cells were stained with the epithelial marker CK and the stromal endometrial marker CD10. Previously, the antibody concentration needed for the detection of both markers was optimized by using primary endometrial epithelial and stromal cells previously cultured. In the optimization process, three different concentrations of the primary antibodies (1:50, 1:150, and 1:250) were tested and two types of negative controls were used: unstained cells and cells stained only with the secondary antibody but not with the primary. After that, the optimal concentration for the primary antibodies was set as 1:100 for CK and 1:250 for CD10. 

The ScreenCell^®^ filters containing the cells were blocked with PBS 0.1% of Tween detergent (Sigma Aldrich, Sant Louis, MO, USA) and 5% of normal goat serum (NGS)(Thermo Fisher Scientific, Waltham, MA, USA) and 5% of bovine serum albumin (BSA) (Sigma Aldrich, Sant Louis, MO, USA) for 30 min at RT. Cells were then incubated for one hour at RT with the primary antibodies against CK and CD10, monoclonal mouse anti-pan-cytokeratin antibody (Santa Cruz Biotechnology, Inc., Santa Cruz, TX, USA) and rabbit anti-CD10 polyclonal antibody (BioNova bioNova científica, s.l., Madrid, Spain), at a dilution of 1:100 and 1:250, respectively. The antibodies were diluted in PBS 0.1% Tween and 3% of NGS and 3% of BSA to reduce background. After incubation with the primary antibodies, cells were washed three times with 1x PBS for 5 min and incubated with the secondary antibodies for 30 min at RT in the dark. Goat Alexa488 anti-mouse (Life Technologies, Carlsbad, CA, USA) and goat Alexa555 anti-rabbit (Life Technologies, Carlsbad, CA, USA) were used to detect CK and CD10, respectively, both in a dilution 1:500. After incubation, filters were washed three times with 1× PBS for 5 min and were mounted on glass slides covered with a 7 mm circular coverslip. ProLong Gold antifade reagent with 6-diamino-2-phenylindole (DAPI, Life Technologies, Carlsbad, CA, USA) was used to detect nuclear DNA. Visualization of the stained cells was performed with an OlympusBX61 microscope (Olympus corporation, Tokio, Japan). All positive cells were counted in the whole area of the filter (7 mm). 

## Figures and Tables

**Table 1:** Patient characteristics and number of circulating endometrial cells in each patient.

Patient Code	Age	Menstrual Cycle Phase	Type of Endometriosis	Stimulation	CECs	
					CK	CD10
1	26	Proliferative	Control	rFSH (antagonist)	0	0
2	31	Proliferative	Control	rFSH (antagonist)	0	0
3	21	Proliferative	Control	rFSH (antagonist)	0	0
4	22	Proliferative	Control	rFSH (antagonist)	0	0
5	37	Proliferative	Endometrioma	rFSH (antagonist)	0	11
6	36	Proliferative	DIE	rFSH (antagonist)	0	5
7	42	Secretory	Endometrioma	HRT	0	17
8	32	Proliferative	DIE	GnRH downregulation + HRT	0	0
9	30	Menstrual	DIE	HRT	0	3
9	30	Proliferative	DIE	HRT	0	57
9	30	Secretory	DIE	HRT	0	22
9	30	Proliferative	DIE	GnRH downregulation + HRT	0	3

The table shows the number of patients included in the study, their age, the phase of the menstrual cycle the date of sample collection, the type of endometriosis (or control patients) and, the treatment that was used in the moment of the blood collection. In addition, the number of CECs found after immunofluorescence is also shown for both tested markers (CK and CD10). CECs: circulating endometrial cells; CK: cytokeratin; CD10: Common Acute Lymphoblastic Leukemia antigen; DIE: deep infiltrating endometriosis; GnRH: gonadotropin releasing hormone; rFSH: recombinant follicular stimulating hormone; HRT: hormone replacement therapy.

**Table 2:** The table shows the studies performed in gynecological diseases (endometrial, breast and ovarian cancer and, endometriosis) [27,28,29,39,40,41,42,43,44,45,46,47,48,49,50,51,52,53]. And it describes which methodology was used for the detection of circulating cells. The table also contains references of reviews specialized on this topic. EpCAM: epithelial cell adhesion molecule; RTqPCR: real time quantitative PCR; ICC: immunocytochemistry; IF: immunofluorescence.

Gynecological Pathology	Benign/Malignant	CTCs/CECs Detection Technology	References
Endometrial cancer	Malignant	EpCAM based; CellSearch^®^ and IF	[41]
		EpCAM based; CellSearch^®^ and IF	[46]
		Density-based, Enrichment (Oncoquick) and RTqPCR	[44]
		RTqPCR and flow cytometry	[43]
		Size-Based Enrichment (Metacell^®^) and Immunodetection	[53]
		RTqPCR	[45]
		Review	[49]
Breast cancer	Malignant	EpCAM based; CellSearch^®^	[27]
		EpCAM based; CellSearch^®^	[29]
		EpCAM based; CellSearch^®^	[28]
Ovarian cancer	Malignant	EpCAM based; magnetic beads	[50]
		ICC	[51]
		Immuomagnetic bead screening and RTqPCR	[47]
		Review	[52]
		Immunomagnetic microspheres	[42]
		Microfluidic system	[48]
Endometriosis	Benign	In culture enrichment; MetaCell^®^	[39]
		IF staining via microfluidic chips	[40]
		ScreenCell^®^	This study

## Abbreviations

CD10	Common acute lymphoblastic leukemia-associated
CECs	Circulating Endometrial Cells
CK	Cytokeratin
COS	Controlled Ovarian Stimulation
CRCs	Circulating Rare Cells
CTCs	Circulating Tumor Cells
DIE	Deep Infiltrating Endometriosis
EMT	Epithelial Mesenchymal Transition
EpCAM	Epithelial Cell Adhesion Molecule
FDA	Food and Drug Administration
GnRH	Gonadotropin Releasing Hormone
IHC	Immunohistochemistry
IF	Immunofluorescence
LGR5	Leucine-Rich Repeat Containing G Protein-Coupled Receptor 5
rFSH	Recombinant follicular stimulating hormone
